# Heparan sulfate mediates trastuzumab effect in breast cancer cells

**DOI:** 10.1186/1471-2407-13-444

**Published:** 2013-10-01

**Authors:** Eloah Rabello Suarez, Edgar Julian Paredes-Gamero, Auro Del Giglio, Ivarne Luis dos Santos Tersariol, Helena Bonciani Nader, Maria Aparecida Silva Pinhal

**Affiliations:** 1 Department of Biochemistry, Universidade Federal de São Paulo, Rua Três de Maio, 100, Vila Clementino, 04044-020, São Paulo, SP, Brazil; 2 Department of Biochemistry, Faculdade de Medicina do ABC, Avenida Príncipe de Gales, 821, Vila Príncipe de Gales, 09060-650, Santo André, SP, Brazil; 3 Department of Oncology, Faculdade de Medicina do ABC, Avenida Príncipe de Gales, 821, Vila Príncipe de Gales, 09060-650, Santo André, SP, Brazil

**Keywords:** HER2, Heparanase, Heparan sulfate, Dermatan sulfate, Proteoglycans, Glycoaminoglycans, Breast cancer resistance

## Abstract

**Background:**

Trastuzumab is an antibody widely used in the treatment of breast cancer cases that test positive for the human epidermal growth factor receptor 2 (HER2). Many patients, however, become resistant to this antibody, whose resistance has become a major focus in breast cancer research. But despite this interest, there are still no reliable markers that can be used to identify resistant patients. A possible role of several extracellular matrix (ECM) components—heparan sulfate (HS), Syn-1(Syndecan-1) and heparanase (HPSE1)—in light of the influence of ECM alterations on the action of several compounds on the cells and cancer development, was therefore investigated in breast cancer cell resistance to trastuzumab.

**Methods:**

The cDNA of the enzyme responsible for cleaving HS chains from proteoglycans, HPSE1, was cloned in the pEGFP-N1 plasmid and transfected into a breast cancer cell lineage. We evaluated cell viability after trastuzumab treatment using different breast cancer cell lines. Trastuzumab and HS interaction was investigated by confocal microscopy and Fluorescence Resonance Energy Transfer (FRET). The profile of sulfated glycosaminoglycans was also investigated by [^35^S]-sulfate incorporation. Quantitative RT-PCR and immunofluorescence were used to evaluate HPSE1, HER2 and Syn-1 mRNA expression. HPSE1 enzymatic activity was performed using biotinylated heparan sulfate.

**Results:**

Breast cancer cell lines responsive to trastuzumab present higher amounts of HER2, Syn-1 and HS on the cell surface, but lower levels of secreted HS. Trastuzumab and HS interaction was proven by FRET analysis. The addition of anti-HS to the cells or heparin to the culture medium induced resistance to trastuzumab in breast cancer cells previously sensitive to this monoclonal antibody. Breast cancer cells transfected with HPSE1 became resistant to trastuzumab, showing lower levels of HER2, Syn-1 and HS on the cell surface. In addition, HS shedding was increased significantly in these resistant cells.

**Conclusion:**

Trastuzumab action is dependent on the availability of heparan sulfate on the surface of breast cancer cells. Furthermore, our data suggest that high levels of heparan sulfate shed to the medium are able to capture trastuzumab, blocking the antibody action mediated by HER2. In addition to HER2 levels, heparan sulfate synthesis and shedding determine breast cancer cell susceptibility to trastuzumab.

## Background

HER2 is a member of the epidermal growth factor family of tyrosine kinase receptors. HER2 is amplified in approximately 14% of breast cancers in early stages and 25% of metastatic breast cancers. HER2 overexpression is associated with lymph node metastasis, short relapse time, poor survival and decreased response to endocrine and chemotherapy [1,2]. Trastuzumab is a humanized, monoclonal antibody that specifically blocks HER2 activation and cell signaling [3]. It is approved for use in patients who have HER2-positive disease, estrogen receptor/progesterone receptor-negative disease or a high-risk feature [4]. Patients that present breast cancer in early stage, when treated with trastuzumab had a 9% increase in absolute disease free survival at five years, while for patients with metastatic disease the period is extended only by five to nine months [2]. Nevertheless, 20% of breast cancer patients in early stages do not respond to trastuzumab therapy and 70% of the patients with metastatic disease who received trastuzumab as monotherapy become resistant to this antibody [5] by mechanisms which are still not completely understood.

Trastuzumab resistance has been investigated and some possible mechanisms have already been described: inactivation of PTEN, since PTEN-deficient tumors have remarkably lower overall response rates to trastuzumab; extracellular HER2 cleavage with p95HER2 formation, which is constitutively activated; HER3 overexpression which implies compensation for HER2 inhibition in cancer cells mediated by trastuzumab; up-regulation of autophagic activity, chaperone action, which may increase HER2 stability or inhibit proteases activity; constitutive activation of crosstalk effectors and finally, the enrollment of extracellular matrix components**,** such as integrins, that can increase signaling pathways for cell proliferation and cellular survival [6,7]. Despite the emphasis placed on the study of molecules responsible for breast cancer resistance to trastuzumab, none of these markers have been proven to be sufficiently reliable to identify patients resistant to this antibody.

Some extracellular matrix components are known to play important roles in tumor development [6]. Heparan sulfate proteoglycans (HSPGs) are essential to cancer cell proliferation, escape from immune response, invasion of neighboring tissues, and metastasis to distal sites. Several tumor types including breast cancers show aberrant modulation of several enzymes related to heparan sulfate (HS)/ heparin biosynthesis, as well as catabolic enzymes such as sulfatases and heparanase-1 (HPSE1) [8,9].

HPSE1 is an endo-β-D-glucuronidase involved in the degradation of both cell-surface and ECM HS in normal and neoplastic tissues. High levels of HPSE1 are associated with metastatic cancers [10]. HS is a reservoir on which heparin-binding growth factors aggregate. Indeed, it has been reported that HS produced by malignant breast cancer cells and the HS oligossacharides generated by HPSE1 possess higher fibroblast growth factor 2 (FGF2) and hepatocyte growth factor binding capacity than HS from normal breast cells [11,12]. Thus, by breaking down HS, HPSE1 releases these signaling molecules, which can promote tumor growth, invasion and angiogenesis [13,14]. Enhanced expression of Syndecan-1 (Syn-1), a cell surface HSPG, may provide a mechanism to restrict FGF action and modulate cell-matrix interactions. Syn-1 shedding is stimulated by HPSE1 and is engaged in tumor progression [10].

In light of all the evidence relating extracellular matrix alterations and cancer development, the present work aimed to elucidate the possible role of HPSE1, heparan sulfate (HS) and Syn-1 in breast cancer cell resistance to trastuzumab. In this work we propose that trastuzumab action is dependent on heparan sulfate to elicit the antibody response.

## Methods

### Cell lines and sulfated glycosaminoglycans

This research was conducted using established cell lines. The study was approved by the Ethics Committee of the “Universidade Federal de São Paulo” (registration number 0645/10), Brazil. Human breast cell lines MCF 10A, MCF7 and SKBR3 were acquired from the American Type Tissue Culture Collection (Manassas, VA). MCF 10A, a non-malignant cell lineage, was maintained in Dulbecco’s modified Eagle’s medium (DMEM)/F12 (1:1, v/v), supplemented with 5% v/v horse serum (Invitrogen, Carlsbad, CA), human recombinant epidermal growth factor (Sigma, St. Louis, MO; 20 ng/ml), hydrocortisone (Sigma; 100 ng/ml), bovine insulin (Sigma; 10 μg/ml), cholera toxin (Sigma; 100 ng/ml), penicillin G (Sigma; 50 U/ml) and streptomycin sulfate (Sigma; 50 μg/ml), at 37°C, 5% CO_2_. SKBR3 cells, which present the highest levels of HER2, and MCF7, with intermediate levels of HER2, (mock-transfected or HPSE1 transfected) were maintained in DMEM supplemented with 10% fetal calf serum (FCS), penicillin G (50 U/ml) and streptomycin sulfate (50 μg/ml)_._ Gentamicin (Affymetrix, Inc, Santa Clara, CA; 400 μg/ml) was added only to pEGFP-N1 HPSE1 transfected cells and G418 only to pcDNA3.1-b (Sigma; 400 μg/ml). Trastuzumab (Herceptin®, Genentech, South San Francisco, CA) was used in different concentrations depending on the assay. Bovine pancreas HS was prepared as described [15,16]. Pig skin dermatan sulfate and shark cartilage chondroitin sulfate are from Seikagaku Corporation (Tokyo, Japan).

### MCF7 cell transfection with HPSE1 cDNA

For MCF7 transfection, a 1.6 kb full-length HPSE1 cDNA, GenBank accession no. AY948074, was cloned into the Eco*RI* and Kpn*I* restriction sites of pEGFP-N1 (Clontech, Palo Alto, CA) and into pcDNA3.1-b (Invitrogen). The HPSE1 cDNA was obtained from MCF7 and demonstrates 99.8% of similarity when compared to the human platelet HPSE1 [17]. pEGFP-N1-HPSE1 or pcDNA3.1-b-HPSE1 was stably transfected into MCF7 using the liposomal transfection reagent FuGENE® 6 (Roche Diagnostics, Indianapolis, IN) according to the manufacturer’s instructions. Stable transfected pEGFP-N1-HPSE1 MCF7 cells were selected with gentamicin for 4 weeks (Additional file 1: Figure S1) followed by green fluorescent protein sorting using flow cytometry (FACSAria, BD Biosciences, Franklin Lakes, NJ). pcDNA3.1-b HPSE1 MCF7 cells were selected using G418, and the use of this clone was restricted to confocal assays to eliminate green fluorescent protein (GFP) interference. Confocal microscopy confirms HPSE1 stable transfection using pEGFP-N1 in the MCF7 cells, as shown in Additional file 1: Figure S1.

### Cell viability assay

Approximately 5.0 × 10^3^ mock-transfected MCF7 (MCF7) and MCF7 containing pEGFP-N1-Heparanase (MCF7-HPSE1), 3.0 × 10^3^ SKBR3 and 1.0 × 10^4^ MCF10A cells were seeded on 24-well plates. Different concentrations of trastuzumab were added the following day. After 3 days, the cells were assayed for 3-(4,5-dimethylthiazol-2-yl)-2,5-diphenyltetrazolium bromide (MTT) (Invitrogen) as described by the manufacturer. The competition assay between trastuzumab and anti-HS antibody (anti-HS mouse IgM clone F58-10E4, Seikagaku Corporation, Tokyo, Japan; dilution 1:50) or heparin 100 μg/mL was performed and cell viability was determined also by MTT on the third incubation day. The assays were performed in triplicate.

### Confocal immunofluorescence assay

9.0 × 10^3^ cells (MCF7, MCF7-HPSE1 and SKBR3) were seeded on coverslips, in the presence or absence of trastuzumab (25 μg/ml) for 72 hours. The cells were fixed with 2% paraformaldehyde/PBS for 30 min, washed three times with 0.1 M glycine/PBS, and permeabilized with 0.01% saponine/PBS for 15 min. Trastuzumab and HS localization were analyzed by incubation with anti-HS-FITC (FITC conjugated anti-HS mouse IgM clone F58-10E4, Seikagaku Corporation, Tokyo, Japan; dilution 1:100) and Alexa Fluor® 594 goat anti-human IgG (1:250) for 1 hour. HPSE1, HER2 and Syn-1 expression were detected using goat anti-heparanase-1 C-20 (Santa Cruz; Santa Cruz, CA, USA), rabbit anti-human erbB2 (Dako Corporation, Carpinteria, CA, USA; dilution 1:350), or mouse anti-human Syndecan-1 (CD138; AbD Serotec, Oxford, UK; dilution 1:100), respectively. The primary antibodies were developed with secondary antibodies conjugated with Alexa Fluor® 350, 488 or 594 (1:250) for 1 hour. Nuclei were stained with DAPI (4',6-diamidino-2-phenylindol; Invitrogen; 20 μg/ml) for 15 min. The coverslips were mounted on microscopy slides with Fluoromont G (Immunkemi, Stockholm, Sweden). Light microscopy analysis was performed with a confocal laser scanning microscope equipped with a Plan-Apochromat × 40 objective under oil immersion (Zeiss, LSM 510 META). The pinhole device was adjusted to capture fluorescence of one airy unit. The images were processed using LSM 510 (Zeiss) and Image J (NIH, Bethesda, MD).

### Fluorescence resonance energy transfer assay

5.0 × 10^3^ MCF7 cells were plated on a 96-well multiwell plate, fixed with 2% formaldehyde in PBS for 30 minutes, washed three times with 0.1 M glycine in PBS, blocked with 1% BSA for 2 hours and incubated with anti-HS 1:100 (Seikagaku Corporation, Japan) and Trastuzumab (25 μg/mL) overnight (ON). The conjugated secondary antibodies Alexa Fluor 546 or 594 (Invitrogen, Carlsbad, CA) were used against anti-HS and trastuzumab, respectively. The excitation spectrum of the cells stained with anti-HS-Alexa Fluor 546 and trastuzumab-Alexa Fluor 594 alone was obtained by excitation of the wells at 543 nm and the emission fluorescence was detected from 550 nm to 680 nm, in intervals of 10 nm wavelengths steps, in the fluorometer (FlexStation3, Molecular Devices, Silicon Valley, CA). The analysis of the interaction between HS and Trastuzumab was made by FRET. MCF7 cells doubly stained with anti-HS-Alexa Fluor 546 and trastuzumab-Alexa Fluor 594 were excited at 543 nm. This wavelength was able to significantly excite only the anti-HS-Alexa Fluor 546, which emits their fluorescence in the range of 594 nm. Therefore, anti-HS-Alexa Fluor 546 is able to excite trastuzumab-Alexa Fluor 594 if both molecules are at a distance of under 5 nm. FRET can be evaluated by calculating the FRET ratio of the fluorescence intensity found in the wavelength of maximum emission of trastuzumab-Alexa 594 (617 nm) over the fluorescence intensity value obtained in the maximum wavelength emission of the Anti-HS-Alexa Fluor 546 (580 nm), as previously described by [18].

### Sulfated glycosaminoglycans analysis

Sixty percent confluent cells, grown on 35 mm culture plates were incubated for 2 days with specific cell culture medium containing 10% FCS in the presence or absence of trastuzumab (25 μg/ml). Afterwards, the medium was removed and replaced with new medium without FCS, containing 150 μCi/ml of carrier free [^35^S]-inorganic sulfate (IPEN, São Paulo, SP, Brazil) in the presence or absence of trastuzumab. After 18 hours the culture medium was removed and the cells washed twice with serum-free medium and detached using 25 mM Tris–HCl, pH 7.4, containing 3.5 M urea. Cell protein was estimated by the Coomassie blue method [19]. The analysis of sulfated glycosaminoglycans (GAG) was performed essentially as described by [20,21]. Protein free GAG chains were prepared from the cellular fraction (cells plus ECM) and culture medium by incubation with maxatase (Biocon Industrial, Rio de Janeiro, RJ, Brazil; 4 mg/ml) overnight, at 60°C. Cells and medium aliquots were submitted to agarose gel electrophoresis in 0.05 M 1,3-diamino propane acetate buffer, pH 9.0, as described by [22]. [^35^S]-sulfate labeled GAGs were exposed to Kodak X-ray film (SB-5), for 2–3 days to identify and quantify each compound. The radioactive bands were scraped from the gel and counted in a liquid scintillation counter (LS 6000 IC; Beckman Coulter Inc., Palo Alto, CA) using UltimaGold™ (PerkinElmer Life And Analytical Sciences, Inc.; Wellesley, MA, USA). The identity of the different GAGs was confirmed by degradation with different lyases: chondroitinases AC and ABC (Seikagaku Kogyo Co., Tokyo, Japan) and heparitinases I and II from *F. heparinum*[15,16,23]. For this assay, the GAGs were precipitated with 3 volumes of absolute ethanol prior to the degradation. The assays were performed in triplicate.

### Quantitative reverse transcriptase polymerase chain reaction (qRT-PCR)

Syn-1, HER2 and HPSE1 mRNA expression were evaluated using MCF10A, SKBR3, MCF7 and MCF7-HPSE1 cells treated or not treated with trastuzumab (25 μg/ml) for three days. Dermatan sulfate glucuronosil-C5-epimerase was analyzed in non-treated MCF7 and MCF-HPSE1 cells. Cells were harvested and total-RNA was extracted using TRIzol reagent (Invitrogen) following the manufacturer's protocol. cDNA was synthesized using ImProm-II™ Reverse Transcription System (Promega, Madison, Wisconsin) and oligo (dT)_12–18_ (Invitrogen), following the manufacturer's protocol. Quantitative PCR analysis was performed using SYBR Green mix (Applied Biosystems, Life Technologies Corporation, Carlsbad, CA) and Qiagen Rotor Gene Q 6000 Detection System (Qiagen, Düsseldorf, Germany). Measurements were normalized against GAPDH and RPL13A geometric mean, using 2^-ΔCt^. The following primers were used: HER2: 5’TGCTGGACATTGACGAGACAGAGT3’ and 5’AGCTCCCACACAGTCACACCATAA3’, HPSE1: 5'TGGCAAGAAGGTCTGGTTAGGAGA3' and 5'GCAAAGGTGTCGGATAGCAAGGG3', and Syn-1: 5’AGGGCTCCTGCACTTACTTGCTTA3’ and 5’ATGTGCAGTCATACACTCCAGGCA3’ and for dermatan sulfate glucuronosil-C5-epimerase: 5’GATCCTCGAGATGAGGACTCACACACGGGG3’ and 5’GATCACCGGTACACTGTGATTGGGAACAAGA3’. GAPDH and RPL13A (60S ribosomal protein L13A) genes were used as internal controls. Primers used to amplify RPL13a were: 5’TTGAGGACCTCTGTGTATTTGTCAA3’ and 5’CCTGGAGGAGAAGAGGAAAGAGA3’ and for GAPDH 5’TCGACAGTCAGCCGCATCTTCTTT3’ and 5’GCCCAATACGACCAAATCCGTTGA3’.

### Degradation of biotinylated HS by HPSE1

HPSE1 action was measured by an ELISA-like method using HS biotinylated [24,25]. MCF7, MCF7-HPSE1 or SKBR3 cells (1 × 10^5^ per well) were cultured in the absence or presence of trastuzumab (25 μg/ml) for 3 days in 60-mm plates. The cells were scraped with 500 μL of sodium acetate 25 mM, pH 5.0, containing protease inhibitors (Invitrogen) and 50 μL of cell extract was incubated with the pre-coated plate, revealed with europium-labeled streptavidin, washed and submitted to 200 μL of enhancement solution (PerkinElmer Life Sciences-Wallac Oy, Turku, Finland). Free europium was measured and the data analyzed in the MultiCalc software (PerkinElmer Life Sciences-Wallac Oy). The product obtained by HPSE1 was expressed by the ratio of degraded HS and total protein from the cellular fraction (μg of HS/μg of total protein).

### Statistical analysis

Statistical analysis was performed using the SPSS® 13 program (SPSS® Inc; Illinois, USA). The variables were tested using the Kolmogorov-Smirnov test. The Mann–Whitney test was used to determine the relation between non-parametric variables. A value of *P* < 0.05 was considered statistically significant.

## Results and discussion

### Breast cancer cell viability in the presence of trastuzumab

**Figure 1:**
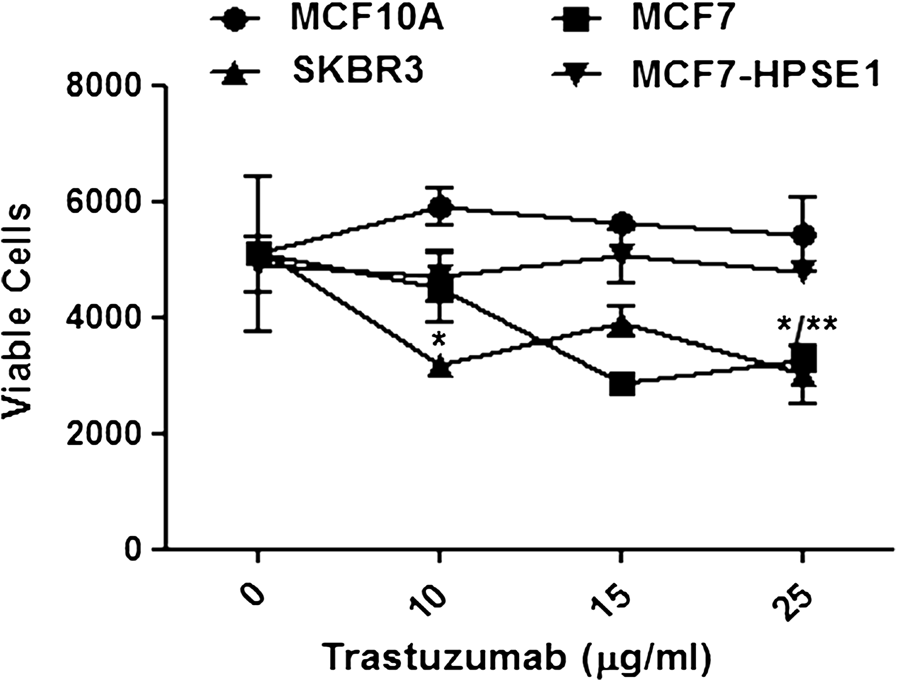
**Cell viability of breast cancer cell lineages treated with trastuzumab.** MCF 10A, MCF7, MCF7-HPSE1 and SKBR3 cells were treated with trastuzumab in different doses for 3 days and assayed using MTT, as described in methods. Each point indicates the mean ± standard deviation (SD) of triplicate assays. *P < 0.05 SKBR3 cells, **P < 0.05 MCF7 cells.

We initially examined cell viability using different concentrations of trastuzumab in MCF10A, SKBR3, MCF7 and MCF-HPSE1 cells. Trastuzumab had no effect on the non-neoplastic MCF10A cells, for all tested doses (Figure 1). Figure 1 shows a decrease of cell viability of around 50% in MCF7 and SKBR3 cells treated with 25 μg/ml of the antibody. SKBR3 is also sensitive to trastuzumab at lower doses. The concentration of trastuzumab that elicits the highest effect in cell viability assays was very similar to the doses used in clinical therapy with trastuzumab (2 mg/kg, to a volume of blood around 0.08 L/kg). Many papers in the literature have shown that at lower doses (up to 3 μg/mL) trastuzumab is not able to decrease the cell viability of MCF7 cells [26-28] probably due to the fact that this cell lineage presents an intermediate HER2 expression. However, an invasion assay using MCF7 cells treated with approximately 8 μg/mL of trastuzumab showed that this antibody was able to inhibit the invasion of these cells in matrigel [29]. Our results showed that trastuzumab in doses higher than 15 μg/mL was able to decrease MCF7 cell viability.

HER2 overexpression by itself is not able to define the response to trastuzumab, since it has already been shown that some cell lineages that overexpress this receptor remain resistant to this antibody [26,30]. These results led us to investigate whether possible differences in breast cancer cell lineages, besides HER2 overexpresssion, could explain trastuzumab resistance.

When MCF7 cells were transfected with HPSE1 cDNA and treated with trastuzumab 25 μg/mL, this antibody lost the ability to decrease the viability of these cells (Figure 1).

### Co-localization between HS, trastuzumab and HER2

**Figure 2:**
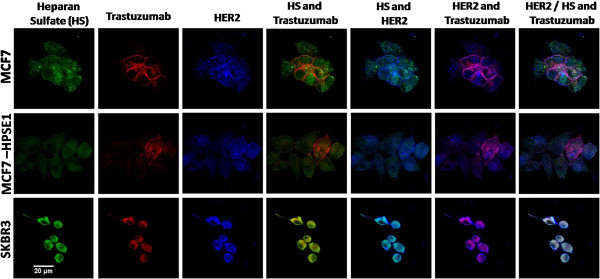
**Co-localization of heparan sulfate, trastuzumab and HER2 in SKBR3, MCF7 and MCF7 HPSE1 cells by confocal immunofluorescence.** 9.0x10^3^ cells were seeded on coverslips and treated with 25 μg/ml of trastuzumab for 72 hours. The cells were fixed with paraformaldehyde, washed with glycine and permeabilized with saponine. The antibodies anti-HS-FITC (represented in green), trastuzumab (stained with Alexa Fluor® 594 – represented in red), and anti-HER2 (stained with Alexa Fluor® 350 – represented in blue) were incubated with the cells as described in methods. Nuclei were stained with DAPI. Microscopy analyses were performed with a confocal laser scanning microscope equipped with a Plan-Apochromat × 40 objective under oil immersion (Zeiss, LSM 510 META). The images were processed using LSM 510 (Zeiss) and Image J (NIH, Bethesda, MD).

Confocal immunofluorescence data demonstrates co-localization between cell surface HS, trastuzumab and HER2 (Figure 2). Table 1 shows the digital quantification of the confocal immunofluorescence analysis, with the mean of intensity and co-localization percentage. The highest co-localization between HS and trastuzumab can be observed in SKBR3 cells (99.5%), followed by MCF7 and MCF7-HPSE1 cells, respectively, 76.2% and 54.7% (Table 1). The same results were obtained by HS and HER2, where SKBR3 and MCF7 presented higher co-localization, 34.8% and 28.1%, respectively, compared to MCF7-HPSE1 (17.2%), as shown in Table 1. As expected, trastuzumab and HER2 were more intensively co-localized in cells sensitive to trastuzumab treatment (SKBR3 and MCF7), compared to the result obtained for MCF7-HPSE1 (Table 1).

**Table 1:** Mean of intensity and percentage of co-localization among HS, Trastuzumab and HER2 by confocal microscopy

	**Mean of intensity (O.D.)**			**Co-localization (%)**		
	**HS**	**Trastuzumab**	**HER2**	**Trastuzumab and HS**	**HS and HER2**	**Trastuzumab and HER2**
**MCF7**	92.3	81.8	102.2	76.2	28.1	29.2
**MCF7-HPSE1**	57.9	50.6	83.4	54.7	17.2	16.2
**SKBR3**	168.6	150.2	179.9	99.5	34.8	35.2

### Interaction between trastuzumab and HS modulates the trastuzumab effect

**Figure 3:**
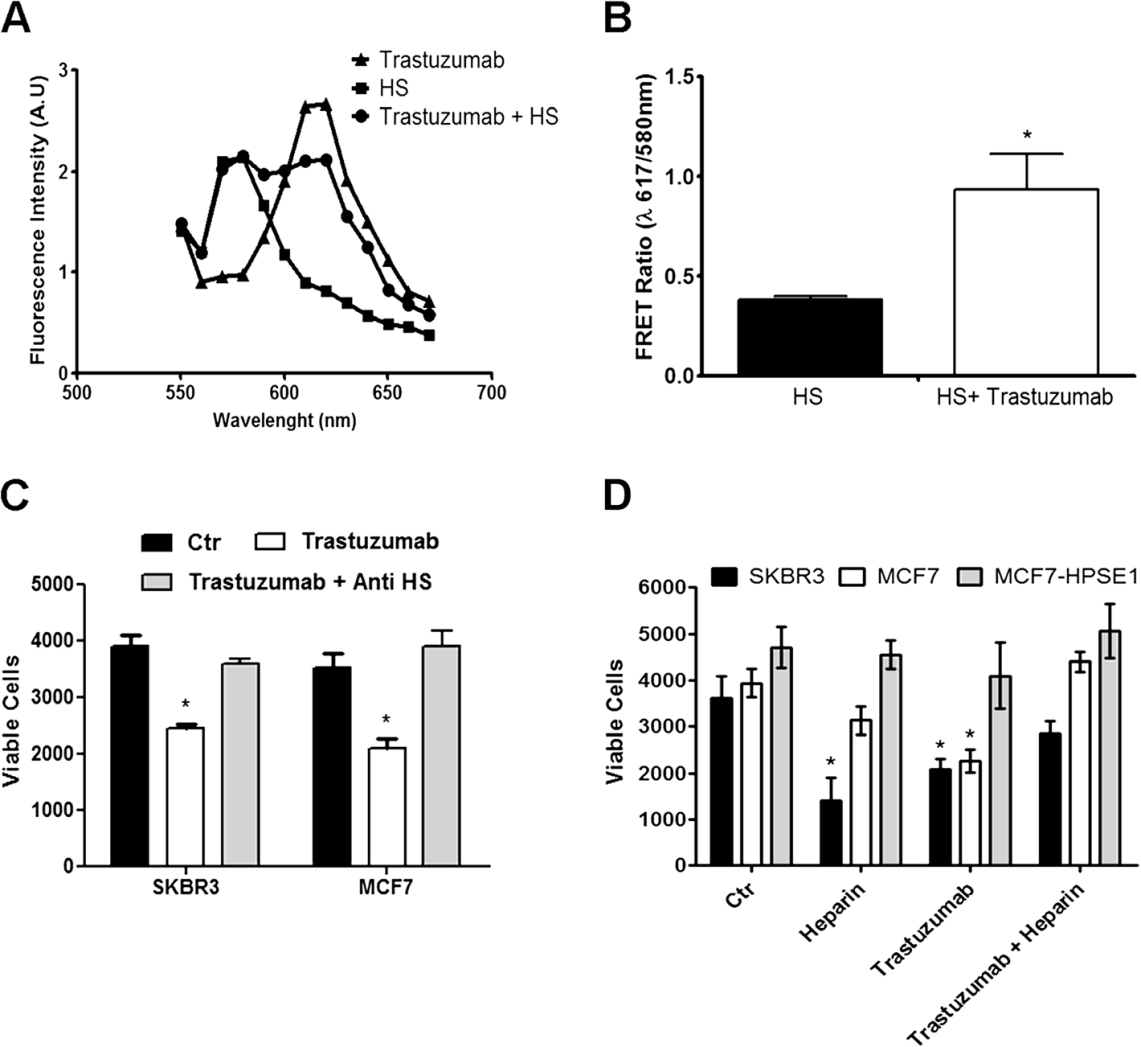
**The importance of cell surface HS to trastuzumab action. (A)**, Emission spectra of MCF7 cells incubated with anti-HS-Alexa Fluor 546 and/or trastuzumab-Alexa Fluor 594 obtained at 543 nm excitation, and fluorescence emission detected from 550 nm to 680 nm, using 10 nm steps in a fluorometer. **(B)**, The values represent the analysis of FRET ratios [ratio of intensity of fluorescence found in the wavelength of maximum emission of trastuzumab-Alexa 594 (617 nm) and the fluorescence intensity obtained in the maximum wavelength emission of the Anti-HS-Alexa Fluor 546 (580 nm)]. **(C)** Reversion of the trastuzumab effect in MCF7 and SKBR3 cell viability assay by an anti-HS antibody. 3.0x10^3^ SKBR3 and 5.0x10^3^ MCF7 cells were treated with trastuzumab (25 μg/mL) and anti-HS antibody 1:100 and cell viability was determined also by MTT on the third incubation day. Each point indicates the mean ± SD of triplicate assays. *P < 0.05, compared to respective control cells. **(D)**, Heparin added to the medium revert the decrease in cell viability induced by trastuzumab in sensitive breast cancer cells. 3.0x10^3^ SKBR3 and 5.0x10^3^ MCF7 and MCF7-HPSE1cells were treated with trastuzumab (25 μg/mL) and/or heparin (100 μg/mL) and cell viability was determined after three days of incubation. Each point indicates the mean ± SD of triplicate assays. *P < 0.05, compared to respective control cells.

FRET assay confirms interaction between HS and trastuzumab (Figure 3A). A 60% increase in FRET ratio was observed when HS and trastuzumab were analyzed in MCF-7 cells (Figure 3B).

A cell viability assay using an anti-HS antibody was performed to confirm whether HS from the cell surface could modulate trastuzumab binding to HER2 (Figure 3C). Anti-HS clearly blocks the trastuzumab effect in MCF7 and SKBR3 cells (Figure 3C). Trastuzumab decreased cell viability by around 40% and this effect was completely reverted by anti-HS (Figure 3C), showing the importance of HS present on the cell surface to elicit the trastuzumab effect.

Heparin, a well-known anticoagulant molecule, is a GAG with a structure very similar to HS. In Figure 3D, heparin was added to the medium of breast cancer cells in order to evaluate the effect of HS/heparin shedding in trastuzumab activity over breast cancer cells. When heparin is co-administered with trastuzumab to breast cancer cells in cell viability assays, it was able to reverse the effect of the monoclonal antibody upon these cells. This result confirms the importance of HS on the cell surface to modulate the trastuzumab effect and also demonstrates the negative impact of HS shedding in the breast cancer cells sensitive to trastuzumab.

It is already known that heparin increased the survival of patients with cancer in randomized trials that compared low molecular weight heparin to unfractionated heparin for the treatment of deep vein thrombosis [31]. Moreover, heparin is able to inhibit the proliferation of some cell types like vascular smooth muscle cells, mesangial cells, fibroblasts and epithelial cells [32,33]. The epithelial lineage used in our experiments, SKBR3, showed sensitivity to heparin, and was able to decrease the viability of these cells by 50% (Figure 3D). Heparin interferes with the activities of growth factors such as FGF-2 and VEGF and blocks selectin-mediated intercellular interactions, inhibiting angiogenesis and tumor development and progression [34,35]. However, heparin can only be administered at relatively low concentrations because of its strong anticoagulant effect [34].

Reversal of the trastuzumab effect on cell viability using an anti-HS antibody that blocks the cell surface HS, or the heparin addition to the medium, mimicking the HS shedding, associated with the interaction between HS and trastuzumab determined by FRET allowed us to prove that trastuzumab depends on cell surface HS to inhibit HER2. As observed, the addition of heparin to the medium inhibits the trastuzumab effect, probably by competing with HS, avoiding the ternary complex formation between trastuzumab, HS and HER2. The crystallography study of HER2 in complex with trastuzumab has shown the presence of a sulfate group and two N-acetylglucosamine residues in the domain I of HER2 [36]. These molecules are important constituents of the heparan sulfate structure. These data, associated with our results, highlight the importance of the HS domains present in HER2 to determine trastuzumab interaction with this receptor and the consequent blocking of the cellular pathways that are over-activated in HER2 positive breast cancer cells.

### Profile of sulfated glycosaminoglycans in breast cancer cells

**Figure 4:**
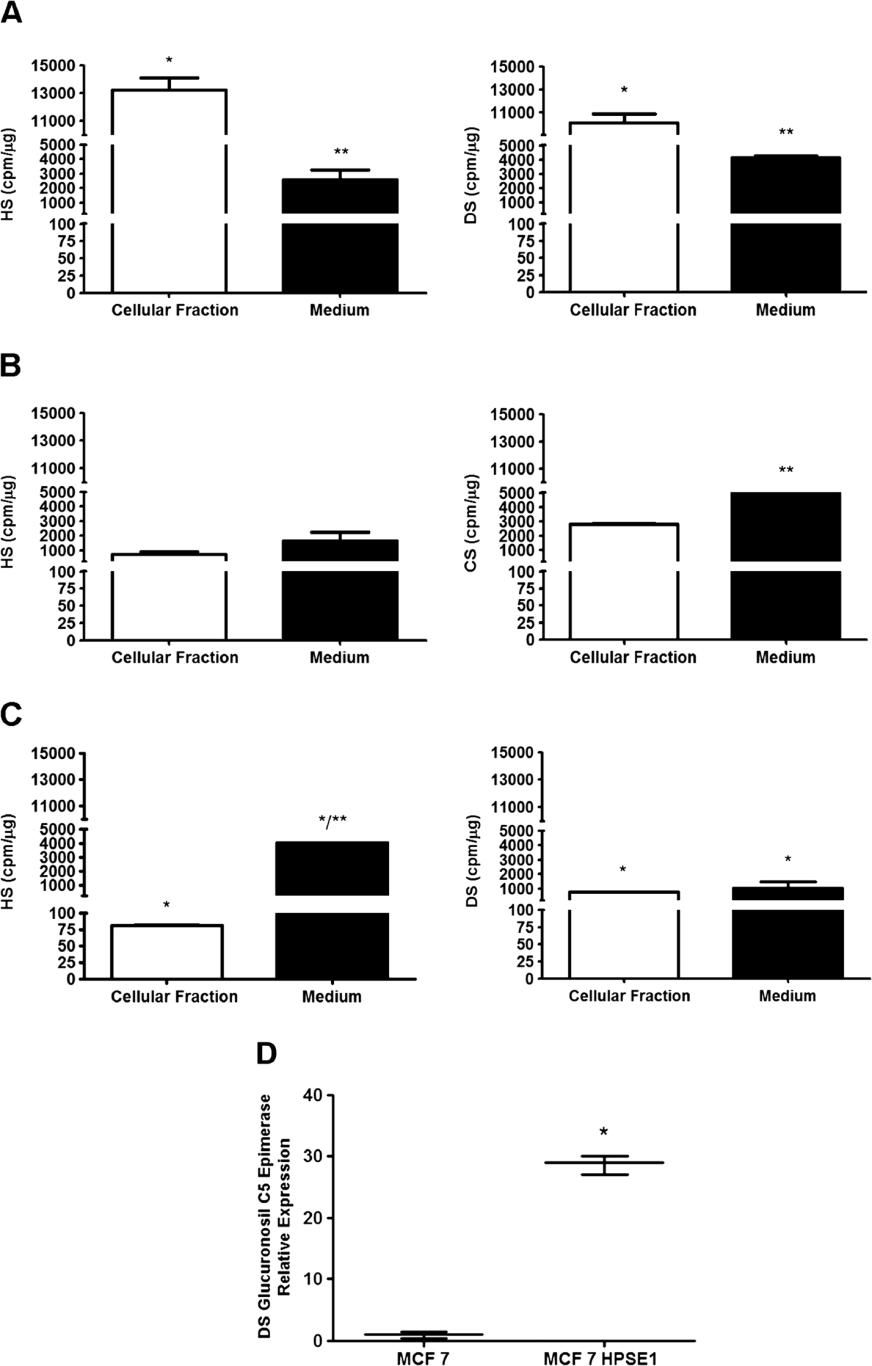
**Glycosaminoglycans profile of SKBR3, MCF7 and MCF7-HPSE1 cells.** Sixty percent confluent cells were incubated for 18 hours in serum-free medium containing 150 mCi/ml [^35^S]-sulphate. Protein-free GAG chains were prepared from the cells and culture medium by incubation with maxatase. Aliquots from the medium and cells were submitted to agarose gel electrophoresis (0.05 M diaminopropane acetate buffer, pH 9.0) and the sulphated GAG identified and quantified as described in methods. **(A)**, Heparan sulfate (HS) and dermatan sulfate (DS) from SKBR3; **(B)**, HS and chondroitin sulfate (CS) from MCF7; **(C)**, HS and DS from MCF7-HPSE1. *P < 0.05, compared to the respective fraction of MCF7 cells, **P < 0.05, medium versus cellular fraction of the same cell lineage. **(D)**, mRNA expression of DS glucuronosil C5 epimerase in MCF7 versus MCF7-HPSE1 cells by qRT-PCR. The RNA was extracted using TRIzol reagent, converted into cDNA by RT-PCR and submitted to qRT-PCR using specific primers for glucuronosil C5 epimerase. The values were corrected by RPL13a (ribosomal protein) and GAPDH expressions. Each bar indicates the mean ± SD of triplicate assays. *P < 0.001.

**Figure 5:**
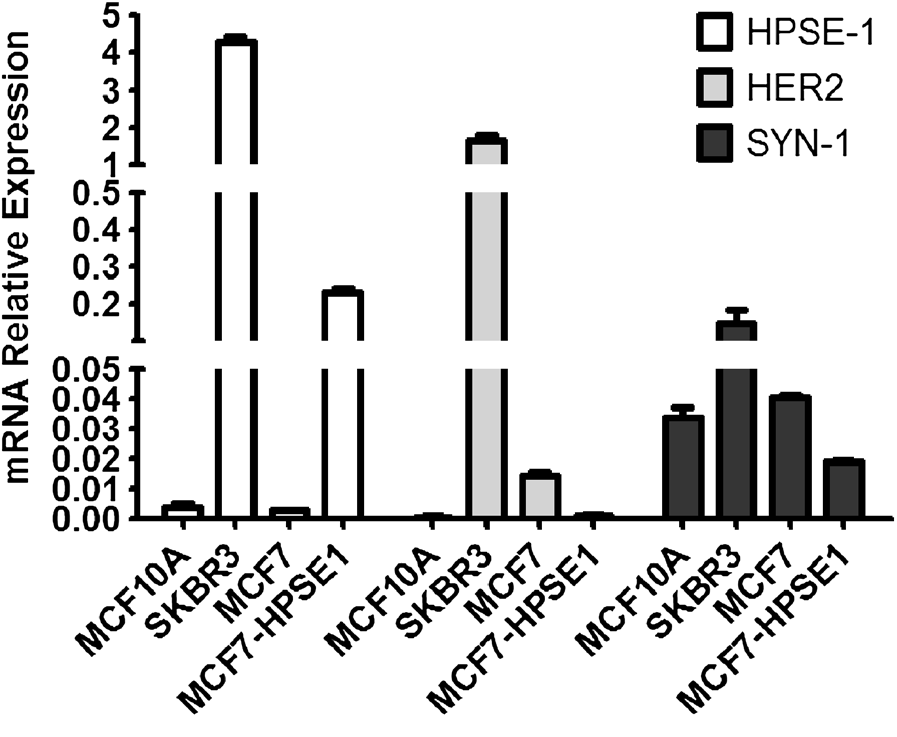
**HPSE1, HER2 and Syndecan-1 mRNA expression in MCF10A, SKBR3, MCF7 and MCF7-HPSE1.** The RNA was extracted using TRIzol reagent, converted into cDNA by RT-PCR and submitted to qRT-PCR using specific primers for HER2, Syn-1 and HPSE1, as described in methods. The values were corrected by RPL13a (ribosomal protein) and GAPDH expressions. Each bar indicates the mean ± SD of triplicate assays. *P < 0.05 compared to MCF10A and **P < 0.001 compared to MCF7.

**Figure 6:**
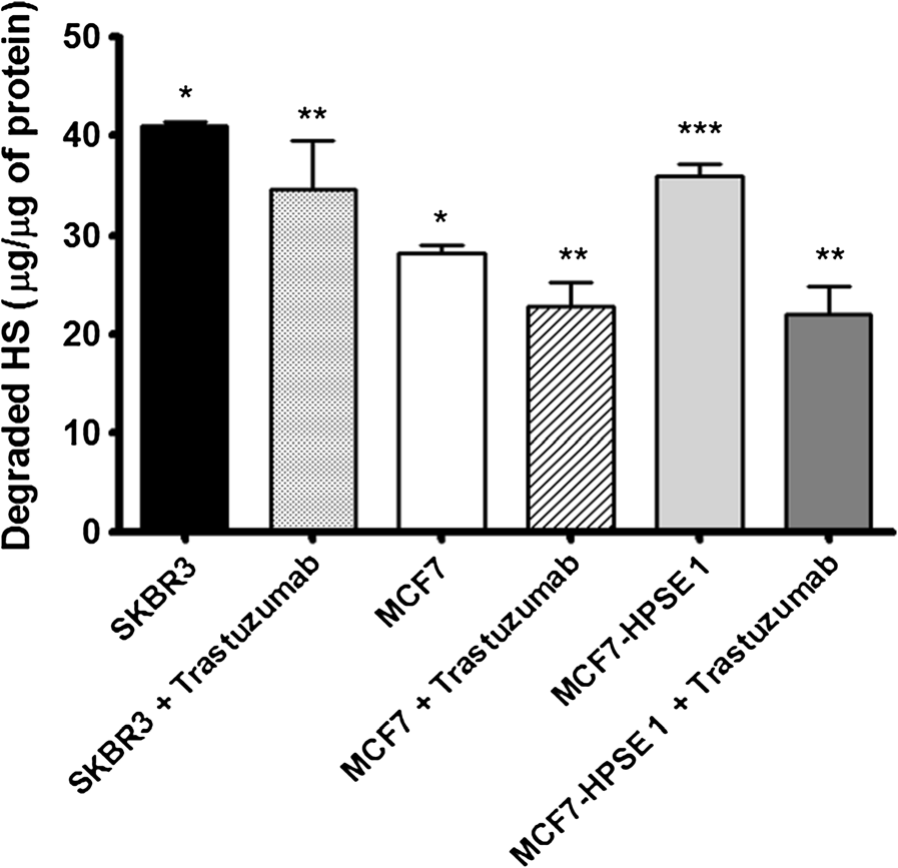
**Degradation of biotinylated HS by heparanase in SKBR3, MCF7 and MCF7-HPSE1 in the presence or absence of trastuzumab.** HPSE1 action was measured by an ELISA-like method using HS biotinylated. Briefly, the cells were cultured in the absence or presence of trastuzumab (25 μg/ml) for 3 days on 60 mm plates. The cells were scraped with sodium acetate 25 mM, pH 5.0, containing protease inhibitors. The cell extract was incubated with the biotinylated HS pre-coated on the plate, revealed with europium-labeled streptavidin, washed and submitted to the enhancement solution. Free europium was measured and the data analyzed using MultiCalc software (PerkinElmer Life Sciences-Wallac Oy). *P < 0.001, compared to the non-degraded biotinylated HS (Control), **P < 0.05, treated *versus* non-treated cells, ***P < 0.05 MCF7-HPSE1 *versus* MCF7. Each bar indicates the mean ± SD. The assays were performed in triplicate.

Figure 4A shows that SKBR3 cells present the highest levels of HS in the cellular fraction (Figure 4B). As shown in Figure 4C, the HS from the cellular fraction in stable transfected MCF7-HPSE1 cells decreased around 14 to 170 fold compared to the MCF7 and SKBR3 cells, respectively. However, similar amounts of HS were secreted to the medium in all three cell lines. It is interesting to note that the value obtained by the ratio of secreted HS/cellular fraction HS was lower in SKBR3, proportional in MCF7 and higher in MCF7-HPSE1. The lower levels of HS secreted in SKBR3 (Figure 4A) is noteworthy, since these cells show high levels of HPSE1 expression and action (Figure 5, Additional file 2: Figure S2 and Figure 6).

Additional file 3: Figure S3 suggests that trastuzumab might be able to induce a retroactive effect in SKBR3 and MCF7 cells, enhancing HS expression in the cellular fraction (Additional file 3: Figure S3A and B). Nevertheless, MCF7-HPSE1 cells treated with trastuzumab showed a decrease in the HS present in the cellular fraction (Additional file 3: Figure S3C). Taken together, the results suggest that HS might modulate trastuzumab binding to HER2, due to increased HS in the cellular fraction of breast cancer cells responsive to trastuzumab in the cell viability assay.

The profile of galactosaminoglycans was determined after degradation with chondroitinases AC and ABC (data not shown). We observed that CS is the galactosaminoglycan present on the cell surface and secreted to the medium of MCF7 cells whilst stable transfected MCF7-HPSE1 cells and SKBR3 expressed DS. Interestingly, DS glucuronosil C5 epimerase, the key enzyme responsible for transforming β−D-glucuronic acid into α–L-iduronic acid [37] is 30-fold overexpressed in MCF7-HPSE1 compared to MCF7, promoting DS synthesis (Figure 4D).

SKBR3 (Figure 4A) presented a 3-fold higher amount of galactosaminoglycans than MCF7 (Figure 4B) in the cellular fraction, but similar amounts of this GAG secreted to the medium. The transfection of MCF7 with pEGFP-HPSE1 was able to promote 3- and 5-fold decreases in the amount of galactosaminoglycan expression in the cellular fraction and medium, respectively (Figure 4C). Curiously, HPSE1 transfection reduces galactosaminoglycans synthesis and changes the profile from CS to dermatan sulfate, related to the increase in the glucuronosil C5 epimerase expression (Figure 4D). Growing evidence suggests that DS plays a crucial role in various biological events such as growth factor signaling, cell division and tumor development [38-41].

### Comparative mRNA analysis of HPSE1, HER2 and Syn-1 in breast cancer cell lines

We also investigated mRNA expression of HPSE1, HER2 and Syn-1 in breast cancer cell lines using qRT-PCR. Figure 5 shows that the mRNA expression of HPSE1 is 3,000-fold higher in SKBR3 compared to MCF10A or MCF7 cells, which present similar HPSE1 mRNA expressions. MCF7 transfected with HPSE1 cDNA showed a 77-fold increase in HPSE1 expression. Since both MCF7 and MCF10A have similar HPSE1 mRNA expression, as shown in Figure 5, and only MCF7 is sensitive to trastuzumab at lower doses, we can surmise that HPSE1 by itself is not decisive in determining breast cancer cell response to trastuzumab. We can also observe in Figure 5 that MCF10A has the lowest expression of HER2 mRNA levels. The HER2 mRNA expression of MCF7 cells is 23 times that of the MCF10A lineage. It is important to point out that despite the low level of HER2 mRNA in MCF7, this cell is not completely negative for HER2. Furthermore, the HER2 mRNA expression of MCF7 was 118 times lower than SKBR3, which corresponds to an established breast cancer cell lineage that presents the highest HER2 levels naturally expressed [42]. Once HPSE1 cDNA was transfected into MCF7 cells, HER2 expression decreased 14 fold, which could contribute to the resistance of MCF7-HPSE1 cells to trastuzumab.

It can be observed that the MCF7 cell line and MCF-10A cells have similar levels of Syn-1 mRNA expression, whilst SKBR3 cells present 5-fold higher Syn-1 expression (Figure 5). When MCF7 cells were transfected with HPSE1, Syn-1 expression decreased 2-fold (Figure 5). The amount of mRNA expression of Syn-1 core protein does not appear to be essential for the response of breast cancer cells to trastuzumab, since MCF7 has similar levels of Syn-1 as the non-responsive MCF10A cells.

### HPSE1 expression and degradation of biotinylated-HS

SKBR3 cells and MCF-7-HPSE1 demonstrated the highest HPSE1 expression and enzymatic action, being able to degrade around 40 μg of HS compared to 20 μg observed in MCF7 cells (Figure 6 and Additional file 2: Figure S2). Furthermore, trastuzumab treatment inhibited HPSE1 action in the breast cancer cells, decreasing HS degradation around 15%-20% in SKBR3 and MCF7 cells and 40% in MCF-7-HPSE1 cells. It has already been shown that MCF7-HPSE1 does not decrease cell viability in the presence of trastuzumab; however, these cells are able to respond to this antibody when other aspects were evaluated. Possibly, the effect of trastuzumab in the inhibition of HPSE1 action in MCF7-HPSE1 cells is achieved even with lower amounts of the complex formed between trastuzumab and HER2. However, the trastuzumab effect upon cell viability probably needs a higher number of complex formations between trastuzumab and HER2 to trigger the molecular signaling that induces cellular apoptosis/necrosis.

### Trastuzumab affects HPSE1, HER2 and Syn-1 expression

**Figure 7:**
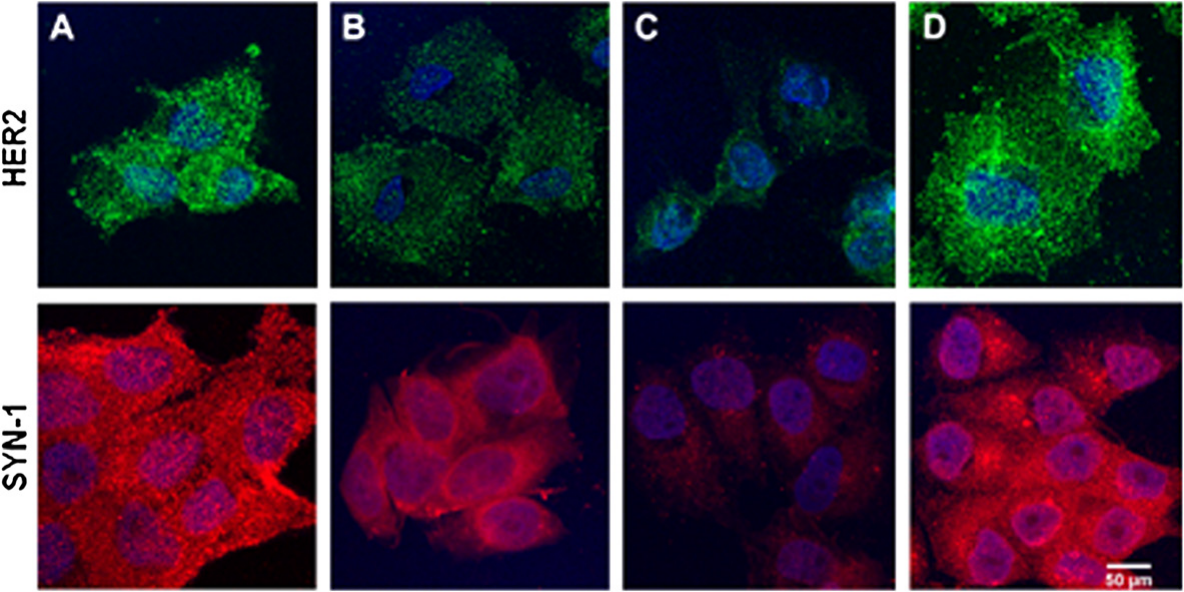
**HER2 and Syndecan-1 expression in MCF7 and MCF7-HPSE1 cells before and after trastuzumab treatment.** These cells were treated for three days with 25 μg/mL of trastuzumab, fixed and prepared for confocal microscopy using the specific antibodies anti-HER2 stained with the secondary antibody Alexa Fluor® 488 (green) or anti-Syn-1 stained with the secondary antibody Alexa Fluor® 594 (red), as described in methods. **(A)** MCF7, **(B)** MCF7 treated with trastuzumab (25 μg/mL), **(C)** MCF7-HPSE1, **(D)** MCF7-HPSE1 treated with trastuzumab (25 μg/mL).

Confocal analysis showed in Figure 7 corroborates with qRT-PCR results previously presented, demonstrating that MCF7 cells have higher amounts of HER2 and Syn-1 proteins on the cell surface (Figure 7A), while stable transfection with HPSE1 cDNA decreases substantially the expression of these molecules (Figure 7C). A decrease in HER2 expression observed in MCF7-HPSE1 before trastuzumab treatment could be related to the resistance against this antibody.

Both SKBR3 and MCF7-HPSE1 overexpress HPSE1. Despite the higher expression and activity of HPSE1, SKBR3 cells compensate HPSE1 overexpression, increasing HS synthesis and Syn-1 mRNA expression, which seems to enhance the susceptibility of this cell line to the effect of trastuzumab on cell viability. However, the MCF7-HPSE1 cell line is resistant to trastuzumab in cell viability assays. Therefore, the compensatory effect observed in the SKBR3 cell is absent in stable transfected MCF7-HPSE1, due to the fact this cell line presents low amounts of Syn-1 and HS on the cell surface.

It has been shown that over-expression of HPSE1 can activate metalloproteases related to Syn-1 shedding [43]. The shedding of syndecan from the cell surface disables cell adhesion, and soluble effector molecules such as growth factors and cytokines bind to HS chains, as they are no longer sequestered on the cell surface. Shedding can impact cell adhesion and growth factor concentration and could act as a stronger promoter of tumor growth *in vivo*[43,44], making MCF7-HPSE1 potentially more aggressive.

A decrease in HER2 and Syn-1 was observed when MCF7 cells were treated with trastuzumab (25 μg/mL) (Figure 7B). Probably the endocytosis induced by trastuzumab can explain decreased HER2 and Syn-1 expression. However, an opposite effect was verified in breast cancer cells resistant to trastuzumab (MCF7-HPSE1), increasing HER2 and Syn-1 protein expression (Figure 7D). It is interesting to note that after HER2 up-regulation by trastuzumab, by an unknown mechanism, MCF7-HPSE1 cells remained resistant to this monoclonal antibody. Therefore, these data indicate that MCF7-HPSE1 trastuzumab resistance in cell viability assays is not dependent exclusively on HER2 expression.

**Figure 8:**
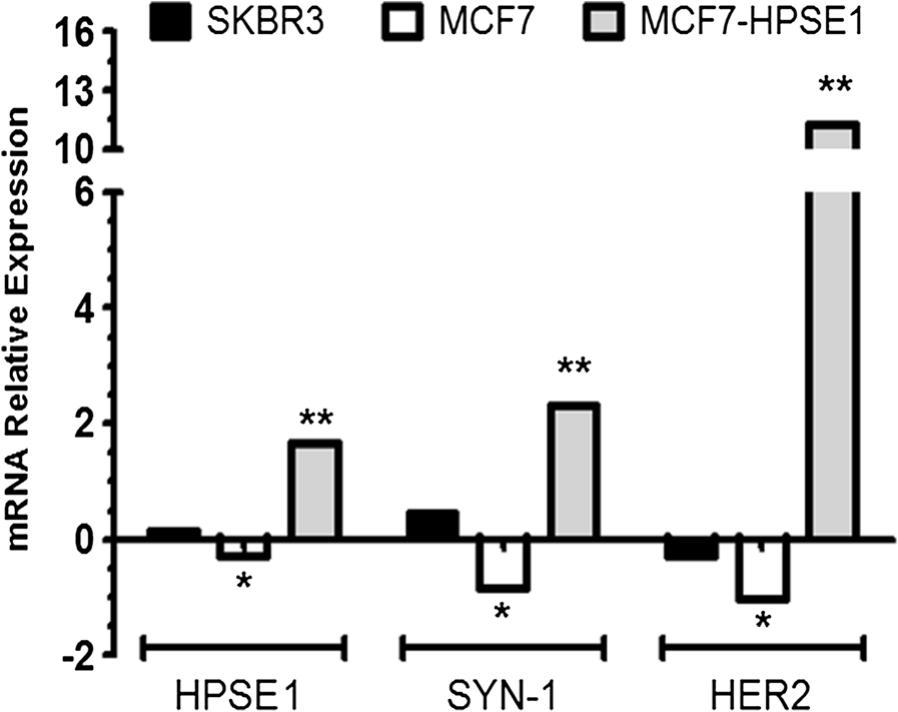
**Ratio of HER2, HPSE1 and Syn-1 mRNA expression of trastuzumab treated per non-treated breast cancer cells MCF7, MCF7-HPSE1 and SKBR3.** These cells were treated for three days with 25 μg/mL of trastuzumab. The RNA was extracted using TRIzol reagent, converted into cDNA by RT-PCR and submitted to qRT-PCR using specific primers for HER2, Syn-1 and HPSE1. The mRNA expression of constitutive genes GAPDH and RPL13A was used to correct the mRNA expression of HER2, Syn-1 and HPSE1, as described in methods. Each bar indicates the mean ± SD of triplicate assays. *P < 0.05 versus non-treated MCF7 cells and ** P < 0.05 versus non-treated MCF7-HPSE1.

The effect of trastuzumab was further investigated by mRNA levels of HPSE1, Syn-1 and HER2. The relative values of HPSE1, Syn-1 and HER2 were obtained by the ratio of mRNA expression after trastuzumab treatment / mRNA expression of non-treated cells (Figure 8). MCF7 cells presented a decrease in mRNA ratio for HPSE1, Syn-1 and HER2 after trastuzumab treatment, indicating that the tumoral phenotype is being reverted when these cells are treated with this antibody. On the other hand MCF7-HPSE1 cells treated with trastuzumab increased the amounts of HPSE1, Syn-1 and HER2 after trastuzumab treatment, which could render MCF7-HPSE1 even more malignant (Figure 8). Despite HER2 upmodulation after trastuzumab treatment, MCF7-HPSE1 remains resistant. If HER2 were the main determinant for trastuzumab effect, MCF7-HPSE1 would become responsive after the treatment.

Despite the presence of HER2, the results obtained so far indicate other differences between trastuzumab sensitive cells (SKBR3 and MCF7), such as higher amounts of cell surface HS and expression of Syn-1, compared to resistant cells (MCF7-HPSE1).

Trastuzumab sensitive cells (SKBR3 and MCF7) synthesize a very significant amount of HS molecules that are maintained on the cell surface compared to the trastuzumab resistant cell line (MCF7-HPSE1), even with higher HPSE1 expression.

Our results suggest that the response of tumor cells to trastuzumab is not only HER2 dependent, but the expression of Syn-1 and particularly the HS present on the cell surface seem to be determinant in trastuzumab action.

## Conclusions

**Figure 9:**
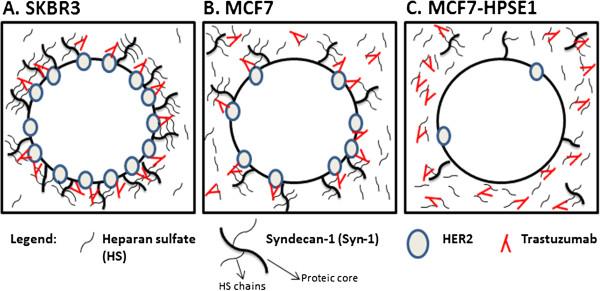
**Molecular profile of breast cancer cells defines trastuzumab efficiency. (A)** SKBR3 cells have the highest levels of HER2, Syn-1, HPSE1 and HS on the cell surface and low levels of HS shed to the medium. This cell is the most sensitive to trastuzumab in cell viability assays. Since HS and trastuzumab interact, as proved by FRET, HS enhances the amounts of trastuzumab available on the cell surface to interact with HER2, facilitating the blocking of this receptor by the antibody. **(B)**, MCF7 cells present intermediate levels of HER2 and Syn-1, low levels of HPSE1 expression and similar levels of HS on the cell surface and shed to the medium, decreasing their viability in the presence of trastuzumab with higher doses. **(C)**, MCF7 cells transfected with HPSE1 cDNA present the lowest levels of HER2 and Syn-1 and high levels of HPSE1. The cell surface HS decreases and the shedding of this molecule increases significantly in these cells. High levels of HPSE1 leads to HS shedding that could capture trastuzumab in the medium, due to the affinity between HS and trastuzumab previously shown. Trastuzumab captured in the media prevents HER2 blocking, contributing to cellular resistance to trastuzumab.

To our knowledge, these are the first results clearly demonstrating an association between a breast cancer cell response to trastuzumab and HER2, HPSE1, Syn-1, HS and galactosaminoglycan synthesis. The results elucidate the importance of ECM components in understanding breast tumor cell resistance to trastuzumab. The hypotheses of the present work are summarized in Figure 9. The amount of HS present on the cell surface has potential as a predictive marker to determine breast cancer patient eligibility for trastuzumab treatment, despite HER2 expression. These new insights could also be useful to develop strategies for overcoming drug resistance in HER2 positive cancers.

## Competing interests

The authors declare that they have no competing interests.

## Authors’ contributions

ERS participated in the conception and experimental design, performed cell viability assays, heparanase activity, glycosaminoglycans analysis, qRT-PCR, immunofluorescence and FRET assays, interpretation of data and drafted the manuscript. EJPG contributed to the design, analysis and interpretation of immunofluorescence assays. ADG contributed to the interpretation of data. HBN and ILST were involved in drafting the manuscript and revising it critically for important intellectual content. MAS was involved in the conception of the assays, interpretation of data, drafting and revising the manuscript and approval of the final version to be published. All authors read and approved the final manuscript.

## Pre-publication history

The pre-publication history for this paper can be accessed here:

http://www.biomedcentral.com/1471-2407/13/444/prepub

## Supplementary Material

Additional file 1: Figure S1 Confocal Immunofluorescence of HPSE1 transfected MCF7 cells (MCF7-HPSE1), using pEGFP-N1 containing HPSE1 cDNA (pEGFP-N1-HPSE1). A 1.6 kb full HPSE1 cDNA, GenBank accession no. AY948074, was cloned into Eco*RI* and Kpn*I* restriction sites of pEGFP-N1 (Clontech). pEGFP-N1-HPSE1 was stably transfected into MCF7 using a liposomal transfection reagent FuGENE® 6 (Roche Diagnostics) according to the manufacturer’s instructions. Stable transfected pEGFP-N1-HPSE1 MCF7 cells were selected with gentamicin for 4 weeks followed by green fluorescent protein sorting using flow cytometry (FACSAria, BD Biosciences, Franklin Lakes, NJ). (A) Nuclear staining with DAPI (blue); (B) recombinant HPSE1 (green), (C) overlapping images. Images captured at 63x magnification under oil immersion (Zeiss, LSM 510 META). Click here for file

Additional file 2: Figure S2 HPSE1 expression by immunofluorescence. HPSE1 expression was detected using goat anti-heparanase-1 C-20 (Santa Cruz). The primary antibody was developed with an anti-goat IgG secondary antibody conjugated with Alexa Fluor® 488 (1:250) for 1 hour. Nuclei were stained with DAPI. (A), Confocal immunofluorescence for HPSE1 in SKBR3, MCF7 and MCF7-HPSE1 cells. Images captured at 40x magnification under oil immersion (Zeiss, LSM 510 META). (B), HPSE1 Intensity of Expression determined by slide densitometry using LSM 510 Software (Zeiss). Click here for file

Additional file 3: Figure S3 Effect of trastuzumab in GAG synthesis and shedding of SKBR3, MCF7 and MCF7-HPSE1 cells. Sixty percent of confluent cells were treated with trastuzumab (25 μg/mL) for 72 hours. In the last 18 hours, cells were incubated with serum free medium containing 150 mCi/ml [^35^S]-sulphate. Protein-free GAG chains were prepared from the cells and culture medium by incubation with maxatase, as described in methods. Aliquots from the medium and cells were submitted to agarose gel electrophoresis (0.05 M diaminopropane acetate buffer, pH 9.0) and the sulphated GAG identified and quantified. (A), Heparan sulfate (HS) and dermatan sulfate (DS) from SKBR3; (B), HS and chondroitin sulfate (CS) from MCF7; (C), HS and DS from MCF7-HPSE1. Each bar indicates the mean ± SD of triplicate assays. *P < 0.05, compared to the respective fraction of non-treated cells. Click here for file
