# Supplementary figures and images for: Heparan sulfate mediates trastuzumab effect in breast cancer cells
Source: BMC Cancer. 2013 Oct 1;13:444. doi: 10.1186/1471-2407-13-444 (PMC3850728; doi:10.1186/1471-2407-13-444)

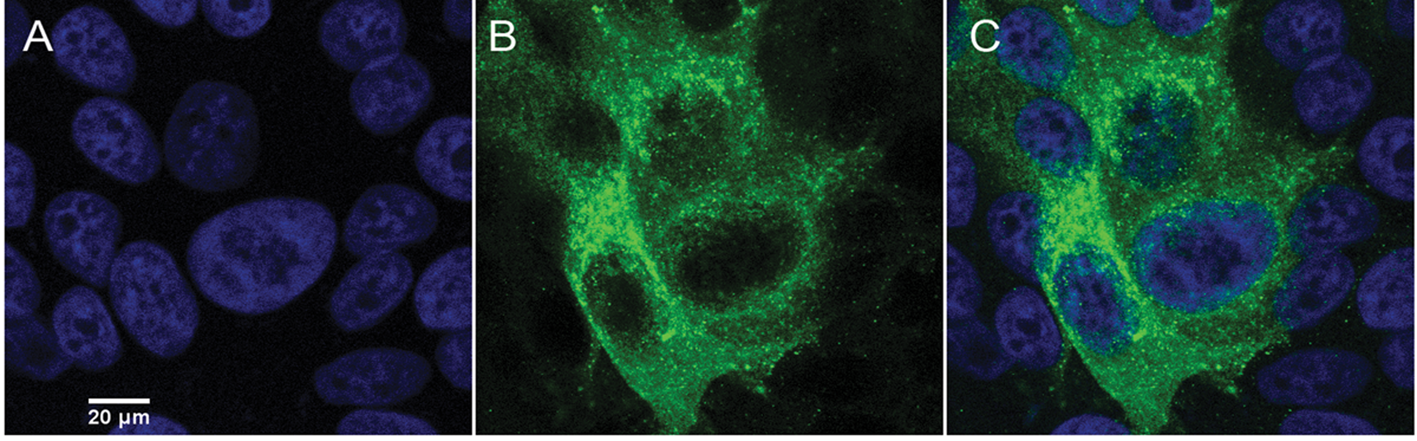

Supplement: Additional file 1: Figure S1 — Confocal Immunofluorescence of HPSE1 transfected MCF7 cells (MCF7-HPSE1), using pEGFP-N1 containing HPSE1 cDNA (pEGFP-N1-HPSE1). A 1.6 kb full HPSE1 cDNA, GenBank accession no. AY948074, was cloned into EcoRI and KpnI restriction sites of pEGFP-N1 (Clontech). pEGFP-N1-HPSE1 was stably transfected into MCF7 using a liposomal transfection reagent FuGENE® 6 (Roche Diagnostics) according to the manufacturer’s instructions. Stable transfected pEGFP-N1-HPSE1 MCF7 cells were selected with gentamicin for 4 weeks followed by green fluorescent protein sorting using flow cytometry (FACSAria, BD Biosciences, Franklin Lakes, NJ). (A) Nuclear staining with DAPI (blue); (B) recombinant HPSE1 (green), (C) overlapping images. Images captured at 63x magnification under oil immersion (Zeiss, LSM 510 META). [file 1471-2407-13-444-S1.tiff]

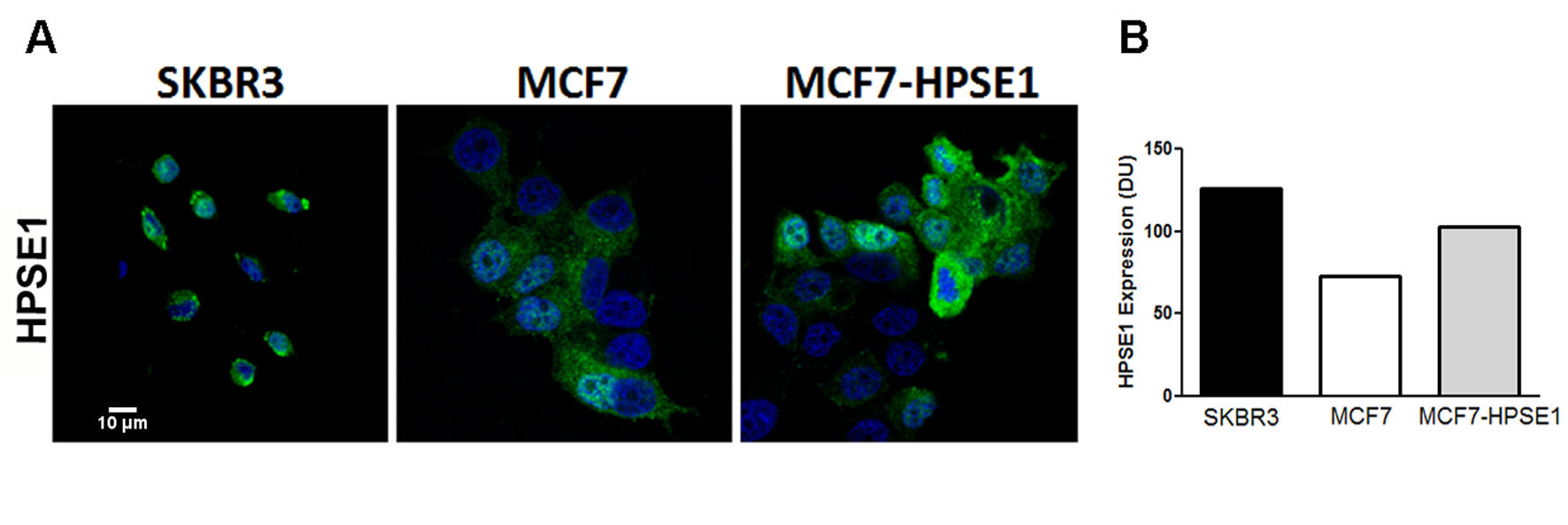

Supplement: Additional file 2: Figure S2 — HPSE1 expression by immunofluorescence. HPSE1 expression was detected using goat anti-heparanase-1 C-20 (Santa Cruz). The primary antibody was developed with an anti-goat IgG secondary antibody conjugated with Alexa Fluor® 488 (1:250) for 1 hour. Nuclei were stained with DAPI. (A), Confocal immunofluorescence for HPSE1 in SKBR3, MCF7 and MCF7-HPSE1 cells. Images captured at 40x magnification under oil immersion (Zeiss, LSM 510 META). (B), HPSE1 Intensity of Expression determined by slide densitometry using LSM 510 Software (Zeiss). [file 1471-2407-13-444-S2.tiff]

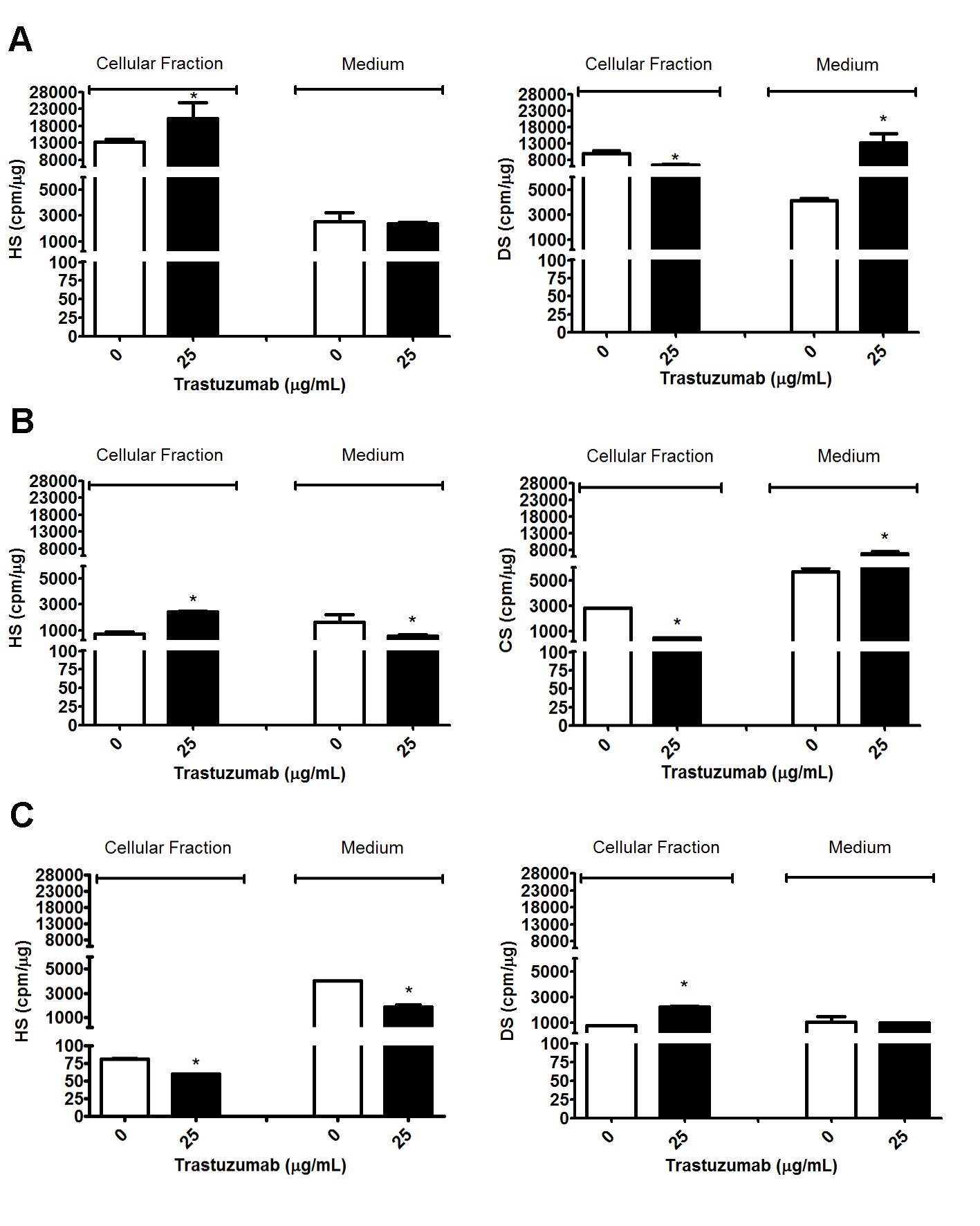

Supplement: Additional file 3: Figure S3 — Effect of trastuzumab in GAG synthesis and shedding of SKBR3, MCF7 and MCF7-HPSE1 cells. Sixty percent of confluent cells were treated with trastuzumab (25 μg/mL) for 72 hours. In the last 18 hours, cells were incubated with serum free medium containing 150 mCi/ml [35S]-sulphate. Protein-free GAG chains were prepared from the cells and culture medium by incubation with maxatase, as described in methods. Aliquots from the medium and cells were submitted to agarose gel electrophoresis (0.05 M diaminopropane acetate buffer, pH 9.0) and the sulphated GAG identified and quantified. (A), Heparan sulfate (HS) and dermatan sulfate (DS) from SKBR3; (B), HS and chondroitin sulfate (CS) from MCF7; (C), HS and DS from MCF7-HPSE1. Each bar indicates the mean ± SD of triplicate assays. *P < 0.05, compared to the respective fraction of non-treated cells. [file 1471-2407-13-444-S3.tiff]
